# Options on fibroid morcellation: a literature review

**DOI:** 10.1007/s10397-015-0878-4

**Published:** 2015-02-07

**Authors:** Hans Brölmann, Vasilios Tanos, Grigoris Grimbizis, Thomas Ind, Kevin Philips, Thierry van den Bosch, Samir Sawalhe, Lukas van den Haak, Frank-Willem Jansen, Johanna Pijnenborg, Florin-Andrei Taran, Sara Brucker, Arnaud Wattiez, Rudi Campo, Peter O’Donovan, Rudy Leon de Wilde, On behalf of the European Society of Gynaecological Endoscopy (ESGE) steering committee on fibroid morcellation

**Affiliations:** 1 Department of Gynaecology, VU University Medical Centre, de Boelelaan 1117, 1181HV Amsterdam, The Netherlands; 2 Department of Obstetrics and Gynaecology, Aretaeio Hospital, St George’s Medical School, Nicosia University, Nicosia, Cyprus; 3 Department of Obstetrics and Gynecology, Aristotle University of Thessaloniki, Thessaloniki, Greece; 4 Department of Gynaecological Oncology, Royal Marsden Hospital, London, UK; 5 Spire Hull and East Riding Hospital, London, UK; 6 Catholic University Leuven, Leuven, Belgium; 7 Donauisar Klinikum Deggendorf-Dingolfing-Landau, Deggendorf, Germany; 8 Leiden University Medical Centre, Leiden, The Netherlands; 9 Elisabeth-twee steden Hospital, Tilburg, The Netherlands; 10 Department for Women’s Health, University Hospital Tuebingen, Eberhard Karls University, Tübingen, Germany; 11 IRCAD, Strassburg, France; 12 Leuven Institute for Fertility and Embryology (LIFE), Leuven, Belgium; 13 M.E.R.I.T. Centre, Bradford Royal Infirmary, Bristol, UK; 14 Pius Hospital, Oldenburg, Germany

**Keywords:** Leiomyoma uteri, Leiomyosarcoma, Endometrial stromal sarcoma, Laparoscopy, Morcellation, Power morcellation, Complication

## Abstract

In laparoscopy, specimens have to be removed from the abdominal cavity. If the trocar opening or the vaginal outlet is insufficient to pass the specimen, the specimen needs to be reduced. The power morcellator is an instrument with a fast rotating cylindrical knife which aims to divide the tissue into smaller pieces or fragments. The Food and Drug Administration (FDA) issued a press release in April 2014 that discouraged the use of these power morcellators. This article has the objective to review the literature related to complications by power morcellation of uterine fibroids in laparoscopy and offer recommendations to laparoscopic surgeons in gynaecology. This project was initiated by the executive board of the European Society of Gynaecological Endoscopy. A steering committee on fibroid morcellation was installed and experienced ESGE members requested to chair an action group to address distinct clinical questions. Clinical questions were formulated with regards to the sarcoma risk in presumed uterine fibroids, diagnosis of sarcoma, complications of morcellation and future research. A literature review on the different subjects was conducted, systematic if appropriate and feasible. It was concluded that the true prevalence of uterine sarcoma in presumed fibroids is not known given the wide range of prevalences (0.45–0.014 %) from meta-analyses mainly based on retrospective trials. Age and certain imaging characteristics such as ‘lacunes’ suggesting necrosis and increased central vascularisation of the tumour are associated with a higher risk of uterine sarcoma, although the risks remain low. There is not enough evidence to estimate this risk in individual patients. Complications of morcellation are rare. Reported are direct morcellation injuries to vessels and bowel, the development of so-called parasitic fibroids requiring reintervention and the spread of sarcoma cells in the abdominal cavity, which may possibly or even likely upstaging the disease. Momentarily in-bag morcellation is investigated as it may possibly prevent morcellation complications. Because of lack of evidence, this literature review cannot give strong recommendations but offers only options which are condensed in a flow chart. Prospective data collection may clarify the issue on sarcoma risk in presumed fibroids and technology to extract tissue laparoscopically from the abdominal cavity should be perfected.

## Introduction

A uterine leiomyoma or myoma is a benign smooth muscle tumour of the myometrium and will be referred to in this article as fibroid. On April 17, 2014, the Food and Drug Administration (FDA) published a press release on the website where the use of laparoscopic power morcellation was ‘discouraged’ due to potential upstaging of uterine sarcoma [1]. The motive to do so was the case history of a patient with a presumed fibroid who underwent a laparoscopic hysterectomy and morcellation of the uterus. The fibroid turned out to be a sarcoma and at reintervention spread of the sarcoma in the abdominal cavity was present. The FDA reported a risk of a uterine sarcoma in patients with presumed fibroids of 0.28 % meta-analysing the data of 18 studies. As some way of morcellation has been used for a long time by gynaecologic surgeons to extract tissue from the vagina or the endoscopic openings in order to enable vaginal or laparoscopic surgery, the professional community was shocked and representatives of many scientific societies published their opinions on the matter [2–9]. A common statement was the lack of solid scientific data to reach strong recommendations with regard to the counselling of patients with fibroids on the issue to choose laparoscopic surgery with its established benefits or for laparotomy to escape the small risks related to morcellation of the fibroid.

This review looks at morcellation complications in cases of presumed fibroids. Although intra-abdominal spread of endometrial cancer, adenomyosis and even trophoblastic tissue by morcellation of the uterus has been reported, these issues will not be addressed in this paper as those diagnoses are less unexpected by their clinical features.

To explore the available literature, the subject will be divided into clinical questions:

- What is the risk of sarcoma in patients with a presumed fibroid
- How to diagnose a uterine sarcoma and distinguish it from a fibroid
- What are the complications of morcellation
- How to prevent morcellation complications
- What are the knowledge gaps
- Recommendations on clinical management in patients with fibroids

The objective of this article is to formulate and grade statements and recommendations on fibroid morcellation based on the level of available evidence. The grading of articles and recommendations was performed according to Eccles et al. [10] (Table 1). As statements can be based on several articles, they are treated as recommendations.

**Table 1:** Grading statements and recommendations [10]

Recommended grade	Evidence
A	Directly based on category I evidence
B	Directly based on: • Category II evidence, or • Extrapolated recommendation from category I evidence
C	Directly based on: • Category III evidence, or • Extrapolated recommendation from category I or II evidence
D	Directly based on: • Category IV evidence, or • Extrapolated recommendation from category I, II or III evidence
Good practice point	The view of the Guideline Development Group
NICE 2002	Recommendation taken from the NICE technology appraisal
Evidence category	Source
Ia	Systematic review and meta-analysis of randomised controlled trials
Ib	At least one randomised controlled trial
IIa	At least one well-designed controlled study without randomisation
IIb	At least one other type of well-designed quasi-experimental study
III	Well-designed non-experimental descriptive studies, such as comparative studies, correlation studies or case studies
IV	Expert committee reports or opinions and/or clinical experience of respected authorities

Adapted from Eccles M, Mason J (2001) How to develop cost-conscious guidelines. Health Technology Assessment 5 (16)

## What is the risk of sarcoma in patients with presumed fibroid(s)

Uterine fibroids are a common disorder with an estimated incidence of 20–40 % in women during their reproductive years [11, 12]. In contrast, leiomyosarcoma of the uterus is a rare entity with an annual incidence quoted at 0.64/100,000 women [13]. According to a recently revised WHO classification of uterine sarcomas, the myometrial pure stromal sarcoma (leiomyoma, Smooth muscle Tumor of Unknown Potential [STUMP] and leiomyosarcoma) is to be distinguished from the endometrial stromal sarcoma (endometrial stromal nodule [ESN], the low-grade endometrial stromal sarcoma and the undifferentiated endometrial sarcoma). The carcinosarcoma or Mixed Müllerian Tumor (MMT) is classified as carcinoma. Excluding the MMT, leiomyosarcoma (LMS) accounts for 70 % and stromal sarcoma for 30 % of all uterine sarcomas [14]. Uterine sarcomas consist 2–7 % of all uterine malignancies. Reliable figures for the incidence of STUMP and cellular fibroids are poorly documented. In one article from a single institution over a period of 36 years, there were 18 cases of STUMP and 72 cases of uterine leiomyosarcoma (none of which had a prior diagnosis of STUMP) in the hysterectomy specimens [15]. All cases of STUMP were registered as disease free after 5 years with only conservative management. Sarcomas spread usually by blood or lymphatic vessels. Five-year survival ranges from 17 to 55 %. Survival of patients with a LMS is strongly associated with the number of mitoses per 10 high power fields (×100 magnification): 1–4, 98 %; 5–9, 42 %; ≥10, 15 %. A LMS embedded and confined to the uterus that is removed ‘en bloc’ is associated with a better survival up to 83 % [14].

### Methods

Two authors (TI, KP) performed a systematic review of the literature on prevalence of uterine sarcoma in patients after uterus surgery, mostly because of presumed fibroids. Searches were performed of Pubmed and Embase using the MESH terms “Fibroids and Sarcoma and uterine neoplasms”, “Myomectomy and Complications”. Further papers were obtained from the reference lists of papers reviewed. Two further papers were submitted for inclusions which were unpublished manuscripts [16, 17] (Ind et al. 2014, personal communication). Only papers with over 500 subjects were included to address the issues of publication bias in smaller series. Further analysis using the terms above and cross referencing with published reviews reference lists on this subject, but with the exclusion of review articles, letters and case reports from the analysis, 12 papers are left where the data seemed reliable and acceptable to help answer the question [8, 16–26]. All evidence finally reviewed has been retrieved from observational single arm cohort studies and is therefore level III evidence. The recommendations are therefore graded as Grade C.

## Results

The overall risk of not previously presumed sarcomatous change in the uterus from all papers was 0.14 % (1 in 700). However, there were large differences between papers with figures varying from 0.49 % (1 in 204) [19] to 0.056 % (1 in 1,788) [16]. On average, papers that looked at myomectomy specimens gave a lower risk of sarcomatous change of 0.08 % (1 in 1,306) compared to those that looked at hysterectomy specimens where the overall pooled risk was 0.15 % (1 in 650). The risk appears to be age related with one study demonstrating a lower risk in patients under 45 years of age (Ind et al. 2014, personal communication).

## Discussion

This meta-analysis, based largely on peer-reviewed articles and two submitted articles, shows a prevalence of 0.14 % which is lower than but within the range of similar literature reviews [1, 6]. In the current selection of studies, the small numbers (<500) were excluded. Recently presented data, submitted for publication, however show that including small trials brings the average reported prevalence of sarcoma down (Pritts E, 2014, personal communication).

Pritts et al. selected 131 articles with 29.877 patients operated for fibroids and found a sarcoma prevalence of 1:7,400 (0.014 %). They explain the large prevalence difference with the available literature by including more prospective trials (50 % half of which randomised trials) that lack the confounder of patient selection of retrospective trials. Also trials with smaller numbers were included increasing the power of the meta-analysis using Bayesian statistics to correct for small patient numbers. Although the large differences in prevalence undermine the credibility of all collected data on the prevalence of sarcoma in presumed fibroids, it is likely that the prevalence is much lower than recently reported. Prospective collection of multicentric data of contemporary patients may clarify the important issue of prevalence.

As the LMS has a similar clinical and diagnostic appearance to the leiomyoma in contrast to the endometrial stromal sarcoma, our interest is primarily to distinguish fibroids from LMS. The endometrial stromal sarcomas usually cause abnormal—non cyclic—uterine bleeding. But also LMS can present as a type 0—100 % protruding in the uterine cavity—causing abnormal uterine bleeding. In the current review, all kinds of sarcomatous changes were included in the meta-analysis, including STUMP’s. This may have blurred the prevalence results. In particular including studies with laparoscopic supracervical hysterectomies, sometimes without the presumption of fibroids, will result in a lower reported prevalence. A difference in prevalence between studies where fibroids intended to be morcellated and the older (pathology) studies where all uteri and fibroids served as a denominator in the prevalence rate has also been demonstrated [27].

Data on age and prevalence does not allow the estimation of an accurate risk of sarcoma in the individual patient scheduled for fibroid surgery but they may be taken into account to define a low and intermediate risk group of patients. The paradox remains that as presumed fibroids are less prevalent in postmenopause, the highest (absolute) number of sarcomas is found in the fourth decade, although the incidence is still extremely low [23]. In one study, 18/21 sarcoma patients (86 %) were premenopausal [28]. The statements of this section are in Table 2.

**Table 2:** Statements on the prevalence of uterine sarcoma in presumed fibroids

Statements	Evidence
The incidence of leiomyosarcoma is 0.64/100,000 per year	
The prevalence of sarcoma in a presumed fibroid is 0.14 % (1:700) with a range from 0.49 % (1:204) to 0.014 % (1:7,400). This large range renders more prospective data collection necessary.	C
The risk of sarcoma in presumed fibroids is positively related to age, although the majority of sarcomas—in absolute numbers—will be in the fourth decade. Below the age of 40 sarcoma in a presumed fibroid is extremely rare.	C
Based on age, an accurate assessment of the risk of sarcoma in patients with presumed fibroids is not possible although a global estimation (intermediate risk versus low risk) could be made	C

## How to distinguish a fibroid from a sarcoma by diagnostic tests

Even if an accurate distinction between fibroids and sarcomas by preoperative diagnostic test is not possible, potential specific characteristics of both disorders would enable the clinician to better predict the presence of sarcoma in a presumed fibroid and counsel patients likewise. Uterine sarcomas are characterised by the common oncologic features such as growth, necrosis and increased vascularity. However, these characteristics occur also in fibroids.

Although the focus of this overview is on fibroid morcellation, the same may be applicable to other therapeutic options such as selective uterine artery embolisation, fibroid ablation, hysteroscopic resection or medical treatment.

In this section, the evidence is reviewed as to the predictive value of imaging and other diagnostic tests in differentiating between a (benign) fibroid and a LMS of the uterus.

### Methods

One of the authors (TvdB) performed a systematic Medline search of the literature in order to map the different diagnostic tests and their characteristics. The following search terms were used: “uterine leiomyosarcoma” [all fields], “diagnosis” [all fields], “ultrasonography” [all fields], “LDH” [all fields] or “LDH isoenzymes” [all fields] and “markers” [all fields]. After including cross references, 37 articles were available for further consideration [22, 28–64].

### Imaging (US, MRI)

There are no pathognomonic features predicting a LMS on any imaging technique [29, 30, 36]. Rapid increase in size (within 3 months) has been reported in case reports of LMS [48, 59] but is generally not distinctive as it may occur in fibroids as well [22]. No growth—in 3 months—may be reassuring unless caused by GnRH [65, 66]. Not only can the uterine sarcoma be accompanied by fibroids responding to GnRH, but it may also be sensitive for estrogen deprivation itself due to its estrogen receptors. It has been reported in a group of 21 uterine sarcomas that all but one (95 %) was either solitary or in case of several myometrial lesions the largest [64]. Another study with ultrasound compared eight LMS and three STUMPs with 225 fibroids and reported that LMSs were significantly larger than other uterine smooth muscle tumours [35]. They were all solitary, and seven of eight lesions had a diameter ≥8 cm. Degenerative cystic changes were observed in four lesions, and increased peripheral and central vascularity was demonstrated in seven lesions. Sensitivity, specificity and positive predictive value of increased central and peripheral vascularity in the diagnosis of LMS were 100, 86 and 19 %, respectively. Combining other sonographic findings with marked central vascularity, the positive predictive value increased to 60 %, but sensitivity decreased to 75 %. 2D ultrasound Power Doppler (USPD) may be related to the nature of the tumour, with a peak systolic velocity having a sensitivity of 80 % for detecting sarcoma with a specificity of 97 %. No studies on sarcoma diagnosis have been published on vascular indices measured by 3D USPD.

Although LMS may have on ultrasound and MRI a similar appearance to fibroids [30, 36], a large >8 cm, solitary, oval-shaped, highly vascularised (peripheral and central) and irregular, heterogeneous myometrial tumour with central necrosis/degenerative cystic changes and absence of calcifications should raise the suspicion of a LMS [29, 30, 35, 41]. MR imaging is superior to CT scan to delineate the extent and to evaluate the tissue characteristics of the lesion [43]. MRI, especially the T2-weighted sequences, may help evaluating tumour extension in the uterus [47] and in differentiating between a leiomyoma and a LMS [51, 54]. In a small series, contrast enhancement after administration of gadolinium (Gd)-DTPA was detected in all 10 LMS, but absent in 28 of 32 uterine degenerated leiomyoma patients [39].

### PET scan

Positron emission tomography has a place in the diagnostic armentarium of presumed fibroids. In PET scanning, a radionuclide (tracer) on a biologically active molecule is visualised. In imaging of fibroids, usually fluodeoxyglucose (FDG) is used, but also other molecules, such as deoxyfluorothymidine (FLT) or alphafluorobeta-estradiol (FES), have been reported. In general, the uptake of FDG in a fibroid is associated with the estrogen status, cellularity and the presence of malignancy [62]. One retrospective study compares different imaging techniques in the case of suspected uterine sarcoma. Of the five sarcomata, all were detected by FDG PET, four by dynamic MRI and two by PowerDoppler ultrasound [58]. FES may be more accurate in distinguishing LMS from fibroids than FDG, with an accuracy of respectively 93 and 81 % [67].

### Serum markers (LDH and CA125)

In a prospective series of 227 patients, the total LDH and LDH isozyme type 3 were elevated in all 10 patients with LMS as compared with degenerated leiomyomas [39]. Elevated CA125 have been reported in patients with LMS, especially in advanced-staged LMS [44, 59]. In a series of 42 consecutive LMS, the values of preoperative serum CA125 were significantly higher in the uterine LMS group than those in the uterine leiomyoma group. However, there was significant overlapping of preoperative serum CA125 between the uterine leiomyoma group and early-stage uterine LMS which limits the clinical use [44].

### Histology

The role of endometrial sampling without abnormal uterine bleeding in the detection of uterine sarcoma is not yet elucidated [68]. In a large series of 938 malignant tumours in hysterectomy specimens from a pathology laboratory, 142 specimens with sarcomas were found of which 72 (51 %) had endometrial sampling. In 62/72 (86 %), the sampling was positive for sarcoma. As preoperative abnormal uterine bleeding was not registered and analysed in this study, it is not clear which patients were selected for endometrial sampling. It may be assumed that abnormal uterine bleeding was the indication for endometrial sampling in sarcoma patients and not the presumed fibroid itself.

Also the role of image-guided *needle biopsies* is not completely clear. The predictive value of a negative biopsy might be expected to be low because of the large areas of necrosis, an excellent negative predictive value is reported using MRI-guided needle biopsies and Bell’s classification on histology [31] with a cut-off level of 2 [45]. With this cut-off level, chosen not to miss malignancy, the sensitivity, specificity, positive and negative predictive values were 100, 98.6, 58 and 100.0 %, respectively. No data are found on the possible spread of sarcoma cells by multiple puncturing of the sarcoma. In breast cancer needle puncture, an increase of positive lymph nodes compared to breast tumours that were not punctured was found [69]. On the other hand, incisional biopsy of melanoma has no apparent effect on tumour spread [70]. Tulandi et al. [55] report on two cases of multiple transabdominal biopsies and frozen section before proceeding with morcellation.

Finally, it should be stressed that even the histological diagnosis of LMS on an intact hysterectomy specimen may be difficult because of locally differing diagnostic criteria [31, 38]. Statements on diagnosis of uterine sarcoma are listed in Table 3.

**Table 3:** Statements on diagnostic tests for uterine sarcoma

Statements	Grade
There are no features predicting a leiomyosarcoma (LMS) on any imaging technique with certainty	C
A large (≥8 cm), solitary, oval-shaped, highly vascularised (peripheral and central) and irregular, heterogeneous myometrial tumour with central necrosis/degenerative cystic changes and absence of calcifications must raise the suspicion of a LMS	D
Rapid increase in size (within 3 months) has been reported in LMS but is generally not distinctive as it may occur in fibroids as well. No growth—in 3 months—may be reassuring unless in combination with GnRH	C
MRI with contrast enhancement may prove helpful in differentiating between LMS and fibroid	C
Total LDH and LDH isozyme 3 may help in differentiating between LMS and fibroid	C
CA125 may be elevated in advanced staged LMS but seems not useful in early stage LMS	C
Endometrial sampling in the detection of uterine sarcoma is indicated in abnormal uterine bleeding. Without abnormal uterine bleeding its role is unclear	D
Transcervical or transabdominal needle biopsy may prove of help in differentiating between LMS and a fibroid, although no data are available on spread of tumour cells caused by the biopsy needle	D

## What are complications of morcellation?

A complication can be defined as an unintended and undesirable event following clinical management resulting in its adjustment or irreversible injury to the patient. Known complications are direct morcellation injuries where the activated morcellator injures intestines or blood vessels. Secondary to morcellation of fibroids, chips can implant on the peritoneum causing parasitic fibroids which may need further surgery. If a presumed fibroid appears to be a sarcoma (or other malignancy), any method of morcellation disrupts the integrity of the tumour, possibly upstaging the disease and affecting survival. In case of power morcellation, the centripetal forces of the cylindrical knife may add to the phenomenon of ‘seeding’ of tumour cells on the peritoneum. Finally, the fragmental state of the specimen due to morcellation may impair proper selection for histologic evaluation of that part of the tumour that is suspicious of malignancy. This might cause treatment delay. In this section, these complications will be discussed separately.

### Direct morcellation injuries

In the field of gynaecology, the majority of symptomatic masses are represented by uterine fibroids. During operative laparoscopy, power morcellation is indispensable to remove large tissue fragments while the laparoscopy offers quicker recovery, less post-operative pain, fewer wound complications and less post-operative morbidity than open procedures. In addition, power morcellators shorten the time of surgery significantly. An ‘electrical cutting device for laparoscopic removal of tissue from the abdominal cavity’ was introduced by Steiner in 1993 [71]. Similar instruments according to the Steiner principle have been commercialised since.

Despite the well-established advantages of power morcellation during laparoscopy, the use of power morcellators is not completely without concern. Milad [72] reviewed the FDA’s adverse event database “MAUDE” between 1992 and 2013, where injuries to the small/large bowel (31), large blood vessels (27), the kidney (3), ureter (3), bladder (1) and diaphragm (1) have been reported using power morcellation. In six cases, the accidents were fatal. This underlines that direct morcellation injuries are serious and, though underreported, extremely rare. It is recommended to maintain adequate distension and use morcellators with a nozzle to promote lateral pealing preventing the morcellator from coring the tissue and thereby losing the morcellator’s tip out of sight [73].

### Parasitic fibroids

Traditionally, parasitic fibroids were thought to be pedunculated subserosal fibroids that were accidentally separated from the uterus and had become attached to another organ in the pelvis for their blood supply (3). The increasing number of case reports of parasitic fibroids after the use of laparoscopic morcellation has contributed to the development of an iatrogenic theory. It is thought that seeding of retained small tissue fragments after morcellation can lead to the development of parasitic fibroids in the peritoneal cavity [74]. Most patients presented with symptoms such as abdominal or pelvic pain, dyspareunia, abdominal distension, urinary frequency and constipation. One of five patients is asymptomatic and the presence of a pelvic mass unexpectedly diagnosed during routine examination or another surgical procedure. Additional published cases indicated that although rare, parasitic fibroids can occur long after laparoscopic morcellation and are often asymptomatic or present with abdominal or pelvic pain. Little is known about the incidence and risk factors of this phenomenon.

To answer questions about the *incidence, risk factors and other characteristics* of parasitic fibroids, one of the authors (HP) conducted a systematic literature search the MEDLINE and Embase databases. The report of this systematic review will be submitted elsewhere.

The overall incidence of parasitic fibroids after laparoscopic surgery with the use of morcellation was reported to be between 0.12 and 0.9 % [75–77]. The reported incidence of parasitic myomas after laparoscopic myomectomy was 0.2–1.2 % [76, 78, 79].

With regards to the *risk factors*, gonadal steroids hormones are known to influence the growth of uterine fibroids. It is hypothesised that prolonged exposure to steroid hormones (e.g. hormonal replacement therapy) during postmenopause could be a risk factor for the development of parasitic fibroids. In case of parasitic fibroids, there is often more than one. Statements on parasitic fibroids are listed in Table 4.

**Table 4:** Statements on parasitic fibroids by previous morcellation

Statements	Grade
The overall incidence of parasitic fibroids after laparoscopic surgery with the use of morcellation is reported to be between 0.12 and 0.9 %	D
The reported incidence of parasitic fibroids after laparoscopic myomectomy is 0.2–1.2 %	D
Premenopausal status and hormonal replacement treatment after primary surgery may be considered as risk factors for the development of parasitic fibroids, however not specific	D

### ‘Upstaging’ uterine sarcoma

Although reducing a uterine sarcoma with electromechanical power morcellation within the abdominal cavity is contrary to oncologic surgical principles, the question remains if sarcoma cells are more inclined to implant in the peritoneum after power morcellation then after ‘en bloc’ removal of the uterine specimen with sarcoma embedded and if survival is affected given the bad prognosis that the sarcoma already has. These two questions have been addressed in literature. Two case reports support the concept of upstaging by power morcellation [80, 81]. In the reported patients, reintervention showed seeding of sarcomatous tissue, which was not visible during initial surgery. These findings have been confirmed in larger studies where the percentage upstaging ranged from 15 to 64 % [18, 28, 82, 83].

Not only electromechanical power morcellation is associated with the risk of upstaging but also other ‘manipulations’ of the sarcomatous tumour, such as myomectomy by laparotomy, subtotal hysterectomy and hysteroscopic resection of submucous fibroids, may affect survival suggesting upstaging [84]. The question as to whether seeding affects survival is also addressed by Seidman et al. [18]. In four of seven patients with LMS who were extracted by power morcellation, seeding was visible during reintervention. Three patients died from the disease and one was alive with metastatic disease. Three patients with morcellated sarcoma without signs of seeding were alive without metastases.

Two studies by Park et al. [85, 86] compared the survival of patients with uterine sarcoma with (*n* = 48) and without morcellation (*n* = 58) during surgery and demonstrated a significant difference of survival in favour of the non-morcellated group (Table 5). Although confounding factors cannot be excluded in these retrospective trials, an effect in favour of the morcellation group would have been expected as clinical suspicion of sarcoma based on size and imaging texture could have resulted in a worse prognosis in the laparotomy group.

**Table 5:** Studies that compared patients operated for uterine sarcoma with and without morcellation. With permission from Nederlands Tijdschrift voor Obstetrie en Gynaecologie [27]

Park 2011 (LGESS)	No.	Age	FU	Recurrence	5 years DFS	ORmv
Morcellation −	27	45.3	64	3/27	84 %	
Morcellation +	23	43.6	66	8/23	55 %	4.03 (1–15)
Park 2011 (LMS)	n	Age	FU	Recurrence	5 years DFS	OR
Morcellation −	31	47.9	52	7/31	65 %	
Morcellation +	25	46.4	27	13/25	40 %	3.11 (1–9)

*LGESS* low grade endometrial stromal sarcoma, *LMS* leiomyosarcoma, *FU* follow-up, *DFS* disease free survival, *ORmv* odds rate mortality risk after morcellation in a multivariate analysis

Recently, a literature review was published by Pritts et al. [87] where she critically appraised the studies that reported on upstaging of sarcoma by morcellation which she evaluated as ‘rather poor’. Although this weakens the alarming statements on upstaging, it does not prove the contrary (the innocence of open morcellation of sarcoma) and underlines the need for further studies. Statements on upstaging of uterine sarcoma by morcellation are listed in Table 6.

**Table 6:** Statements on the complication of morcellation ‘seeding’ (upstaging uterine sarcoma)

Statements	Grade
The quality of research regarding upstaging of uterine sarcoma by open morcellation is rather poor	D
Electromechanical power morcellation of an unsuspected uterine sarcoma may cause intraperitoneal dissemination (‘seeding’)	C
Intraperitoneal dissemination (‘seeding’) may be associated with lower survival rates	C
‘En bloc’ resection of a uterine sarcoma may be associated with better survival than other tissue retrieval methods going with tumour injury	D

### Missing the diagnosis of malignancy because of shredded material

Morcellated specimens are poorly amenable to pathologic examination because the morcellation abolishes many of the anatomic features that allow meaningful gross description, including the notions of orientation, dimension, adjacency, border and margin [88]. This has been described in other uterine malignancies and could lead to delayed diagnosis or suboptimal staging, causing treatment delay [89, 90].

## How to prevent morcellation complications

Given the scarcity of direct morcellation injuries, no clinical trials are available and all suggestions to prevent them may be considered as good practice points. With regards to preventing direct morcellation injuries, the options are listed in Table 7; regarding the development of parasitic fibroids, the options are mentioned in Table 8. The options to prevent upstaging of uterine sarcomas by power morcellation will be presented under the final options in Table 10.

**Table 7:** Options to prevent direct morcellation injuries

Options	Grade
For safe entry, enlarge the skin and fascia incision to the diameter of the morcellator to reduce the abdominal wall resistance	Good practice point
Make sure that the morcellator’s blade remain locked inside the protecting tube during the morcellator insertion into the abdomen	Good practice point
Keep the tip of the morcellator shaft in midline of the lower abdomen while introducing the device into the abdominal cavity and during morcellation	Good practice point
Morcellate only under continuous vision by applying the lateral pealing technique. Prevent penetrating the mass and losing the tip out of sight	Good practice point
Morcellation close to the intestine or to blood vessels increase risk of injury to these structures	Good practice point

**Table 8:** Statements and options on preventing parasitic fibroids after morcellation

Statements and options	Grade
The small risk of parasitic fibroid with laparoscopic morcellation (<1 %) should be discussed with the patient and balanced against alternative treatment options	Good practice point
Avoid spread of cells and tissue fragments in the abdominal cavity by stabilising the specimen and prevent fast rotation	Good practice point
When morcellation is used, efforts should be made to prevent tissue loss during morcellation and to remove all tissue fragments after morcellation: Place the patient in reverse Trendelenburg position after morcellation and irrigate the abdomen and pelvis extensively After irrigation of the peritoneal cavity the abdomen and pelvis should be inspected to identify any remaining tissue fragments	Good practice point
The potential increased risk of parasitic fibroids after sex steroid exposure (endogenous/exogenous) after laparoscopic morcellation should be considered before hormonal replacement therapy is prescribed	D

## Knowledge gaps

The—on some issues—systematic literature search on which this review is based revealed some serious gaps in available knowledge. Due to the low prevalence of uterine sarcomas, most data on prevalence and risk factors are derived from case histories or retrospective trials with low numbers. Prospective data collection of patients after fibroid surgery on age, imaging and laboratory results and subsequent histology is needed to answer questions on individual risk estimation in order to enable the patient to give a well-considered consent prior to minimal invasive surgery.

The development of safer morcellation techniques in the abdominal cavity by technical innovation, including in-bag morcellation, is in its infancy. No doubt that safe in-bag morcellation of fibroids has the potential to avoid many of the reported morcellation complications, such as direct morcellation injuries, parasitic fibroids and the upstaging of morcellated sarcomas. However, not all risks are addressed such as spillage from the content of the bag in the abdomen especially if the bag is punctured to introduce a laparoscope in the bag for better visualisation of the morcellator’s tip. The in vitro results are promising. Cohen and Einarsson [91] demonstrated *in-bag* morcellation in an in vitro study, in which they successfully morcellated beef tongue specimens. In only 1 of the 13 trials did leakage of the bag occur. Washings of the container after retrieval of the specimen bag were negative for muscle cells, except for the open control and the trial with leakage of the retrieval bag. A small series of in-bag morcellation have been published with good results [92, 93]. Vaginal morcellation in a bag has been described also [94]. Possible draw backs are the need for a sufficient vaginal entry and the fact that only hysterectomy specimens are suitable for this technique. Future studies must establish the role of vaginal in-bag morcellation.

In urology in-bag (‘contained’) morcellation has been performed in low-grade renal cell carcinoma (RCC). In a retrospective study, Wu et al. [95] evaluated in-bag morcellation in 188 patients with low-stage RCC. After a mean follow-up of 21 months, no difference in survival was demonstrated compared to open nephrectomy, although one port site metastases occurred. The safety and effectiveness in terms of survival was confirmed in another study [96]. In low-grade renal cell carcinoma, laparoscopic approach combined with *in-bag* morcellation of the kidney appears to be safe and effective.

Port site metastases after morcellation are thought to be related to the laparoscopic approach itself and not to the morcellation technique as exteriorising the bag before morcellation is meant to prevent contact of the tumour with the abdominal wall. In a review on port site metastases, the in-bag morcellation technique is recommended to prevent port site metastases [97].

Two studies report on the techniques of containment. Parekh et al. [98] suggested that specimens should be morcellated in fluid-filled retrieval bags. In an in vitro study, five porcine kidneys were morcellated. No perforation or leakage occurred in the fluid-filled bags compared to one perforation in ‘dry’ morcellation. Also, morcellation time was shorter, when compared to the dry bags.

Meng et al. [99] suggested that washings of a specimen retrieval bag may help the pathological diagnosis after renal morcellation. In their prospective study, 22 consecutive washings were examined. In 9 of 13 patients with carcinoma, the cytological examination confirmed pathology and in three cases cytology provided additional information. In all nine benign cases, cytology was consistent with pathology.

The potential benefits and risks of in-bag morcellation should be further evaluated by clinical studies before it can be recommended in general practice. Furthermore, although in-bag morcellation appears to be very promising as a tool for the prevention of morcellation-related complications, further research is needed to improve the morcellation mechanism itself [100]. The statements on knowledge gaps and potential technical innovations are listed in Table 9.

**Table 9:** Statements on technical innovation

Statements	Grade
Research on technical innovation in tissue retrieval from the abdominal cavity mainly focusses on in-bag (‘contained’) morcellation	D
In-bag morcellation may prevent morcellation complications such as direct morcellation injuries, parasitic fibroids and upstaging eventual malignancies	Good practice point
Potential reported risks of in-bag morcellation is spillage of tumour cells from the bag	C
In urology in-bag morcellation after laparoscopic removal of early stage and low grade renal cell carcinoma is reported to be safe and effective	C
Vaginal in-bag morcellation has also been described and needs further study	D
Development of bags is needed as well as registration of cases to further establish the potential value of on in-bag morcellation in gynaecologic surgery	Good practice point

## Concluding remarks

In the previous sections, the results of a literature search are shown to answer questions about fibroid morcellation in laparoscopy, related complications and what is needed to prevent them. It has not been the aim of this study to address morcellation of other structures such as uteri without presumed fibroids (e.g. some cases of LSH) or morcellation of ovarian masses.

**Fig. 1:**
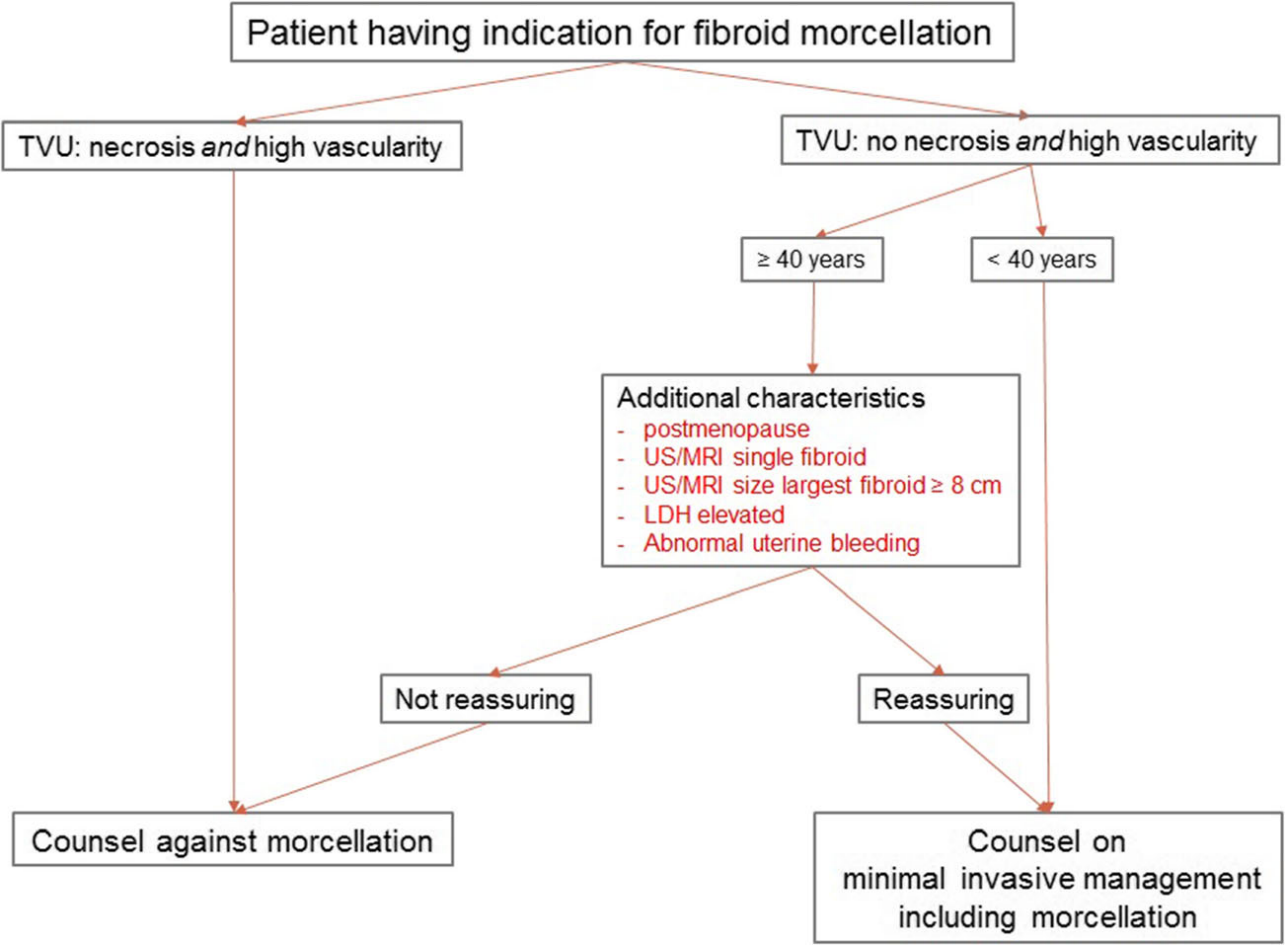
Flowchart of intended fibroid morcellation

As expected, the level of evidence is not sufficient to give recommendations. Therefore, it was decided to present options (Table 10), which are condensed in a flowchart to offer structure in the clinical management (Fig. 1). The flowchart can at best support the clinician to pursue safe and effective fibroid treatment but ‘reassuring’ does not exclude sarcoma and in ‘non-reassuring’ cases the diagnosis will likely be benign. Therefore, it cannot have the status of guideline.

**Table 10:** Options in intended fibroid morcellation

Options and considerations	
Informed consent by the patient is the corner stone of preoperative workup. If fibroid morcellation is intended, include its possible complications in the informed consent procedure before operation.	Good practice point
Standardise the clinical management by using a flowchart to classify patients according to global risk of a sarcoma in a presumed fibroid. Use flowchart in the figure as an option.	Good practice point
Use transvaginal ultrasound, transabdominal ultrasound or in case of poor visualisation on ultrasound MRI with or without contrast (Gadolinium-DTPA)	D
Consider including vascularity parameters (RI and PSV in 2D PowerDoppler ultrasound (PDUS) or vascular indices in 3D PDUS)	D
Consider performing LDH and iso-enzyme 3 assay	D
Perform a preoperative endometrial aspiration in case of abnormal uterine bleeding	D
Support the development of potentially beneficial techniques to prevent morcellation complications by participating in clinical trials	Good practice point
Register patient’s data after her consent including pre-surgery images and post-surgery histology	Good practice point

The ‘additional characteristics’ in the flowchart for women over 40 years of age are based on the literature review in the previous sections. These characteristics have been associated with discrimination of sarcomas from fibroids but they lack thorough scientific evaluation. In the case of LDH, the predictive value is based on only one trial [39]. We have to realise we are looking for a needle in a haystack indeed. This means that for each single ‘risk factor’, we can easily argue that it is of no importance. However, the association of several risk factors is probably more relevant than a single one. If more risk factors are present, it might be wise to be prudent, although the risk of sarcoma remains low.

Fibroid growth has been discussed among the authors at length and it was concluded that ‘growth’ or ‘rapid growth’ are not specifically associated with sarcoma but also with fibroids. On the other hand, a stable size during several months without administration of suppressing hormones (GnRH or Ullipristal) does make a sarcoma highly unlikely. Growth of the presumed fibroids during GnRH treatment or in menopause should raise suspicion about the nature of tumour.

The in-bag morcellation of presumed fibroids has the potential to prevent the rare morcellation-related complications typical of ‘open’ morcellation, such as direct morcellation injuries by distending the morcellation bag, the spread of fibroid chips, sarcoma or other malignant particles and cells in the abdominal cavity. More research is needed to improve the technique and on safety before in-bag morcellation can be recommended as a general tool in laparoscopic surgery.

We have chosen not to list all the literature on the benefits of laparoscopic surgery which are an important argument in the trade-off between laparoscopy with morcellation and laparotomy without. The vast amount of evidence in favour of laparoscopy is elegantly abstracted in the ‘second look’ article by Pitkin and Parker [101].

Taking needle biopsies before surgery would be an interesting option in case of intended fibroid surgery to rule out sarcoma; however, there are different opinions on the potential harmful effects of tumour manipulation and spread by needle biopsy [45, 69, 70]. Also representativeness of needle biopsies may be a yet unresolved drawback, given the necrotic areas in the sarcoma.

It is clear that there is much research to be done. At first, the issue on sarcoma risk in patients scheduled for fibroid morcellation should be clarified. The preliminary report of Pritts et al. (2014, personal communication) that includes more prospective trials and is based on sound statistics shows much lower prevalences (1:7,400) than previously reported in the meta-analyses based on retrospective trials (1:352) [1].

Secondly, more data must be collected to estimate the risk of sarcoma in individual patients with presumed fibroids, based on epidemiological data from the patient and diagnostic tests such as imaging.

Thirdly, technical innovation, such as in-bag morcellation, should enable safe morcellation of intra-abdominal specimens.

A corner stone in the doctor and patient relationship is the informed consent procedure. This means that the patient needs to be informed as completely as possible and based on the best available evidence in order to let her make the best choice of treatment tailored to her individual situation.
